# pH-Mediated Antibacterial Dyeing of Cotton with Prodigiosins Nanomicelles Produced by Microbial Fermentation

**DOI:** 10.3390/polym9100468

**Published:** 2017-09-23

**Authors:** Jixian Gong, Yanfei Ren, Ranran Fu, Zheng Li, Jianfei Zhang

**Affiliations:** 1 School of Textiles, Tianjin Polytechnic University, Tianjin 300387, China; tjpuryf@126.com (Y.R.); frrhappy@163.com (R.F.); lizheng_nx@163.com (Z.L.); 2 Key Laboratory of Advanced Textile Composites, Tianjin Polytechnic University, Ministry of Education, Tianjin 300387, China

**Keywords:** pH-mediated dyeing, antibacterial property, prodigiosins, nanomicelles, microbial pigment, cotton

## Abstract

This study developed a novel pH-mediated antimicrobial dyeing process of cotton with prodigiosins nanomicelles produced by microbial fermentation. The average diameter of the pigment nanomicelles was 223.8 nm (range of 92.4–510.2 nm), and the pigment concentration was 76.46 mg/L. It was found that the superior dyeing effect of cotton fabric was achieved by adjusting the dye bath pH. When the pH was three, dyed cotton under 90 °C for 60 min exhibited the greatest color strength with good rubbing, washing and perspiration color fastness. By the breaking strength test and XRD analysis, it was concluded that the cotton dyed under the optimum condition almost suffered no damage. In addition, due to the presence of prodigiosins, dyed cotton fabric under the optimal process showed outstanding bacteriostatic rates of 99.2% and 85.5% against *Staphylococcus aureus* and *Escherichia coli*, respectively. This research provided an eco-friendly and widely-applicable approach for antimicrobial intracellular pigments with the property of pH-sensitive solubility in water to endow cellulose fabric with color and antibacterial activity.

## 1. Introduction

In recent years, deep development and application research of antimicrobial materials are attracting more and more attention and interest of researchers [1,2,3]. As the most abundant and widespread natural polymer in nature, cellulose is widely applied in textile, medicine and health, paper, food and other industries. The efficient antibacterial modification or finishing of cellulose is always the target sought by people [4,5,6]. During this process, dyeing cellulose with the antimicrobial dyestuff is an effective and interesting method, which could not only bring an antibacterial property, but also impart apparent color to cellulose [7,8].

**Figure 1:**
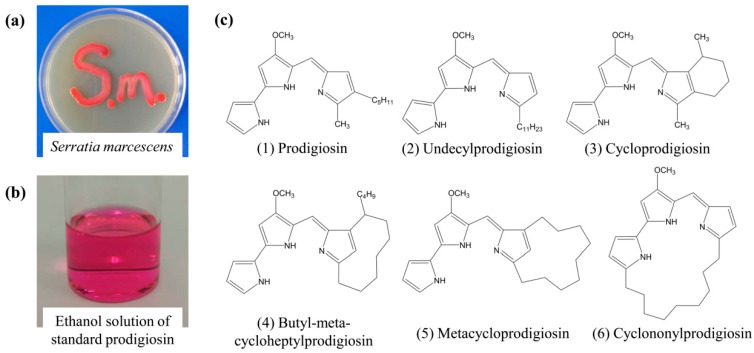
(**a**) *Serratia marcescens*; (**b**) ethanol solution of standard prodigiosin; (**c**) chemical structures of the representative members of prodigiosins family.

With the aggravation of environment and energy problems, people have made more efforts in developing eco-friendly, renewable and healthy resources for the production of dyestuff. At the same time, since many kinds of natural dyes possess antimicrobial and other valuable properties, deep development of natural dyes has become the general trend in the application domain of dyestuff [9,10,11]. Among natural dyes, microbial pigments have gradually become a research hotspot on account of their rich species, short production cycle and high productivity. During the exploration, prodigiosins have had more attention paid to them for their bright color, excellent antimicrobial activity and relatively good thermostability. Prodigiosins are the secondary metabolites of *Serratia marcescens*, *Vibrio gazogenes* and some actinomycetes. As shown in Figure 1, they are a family of microbial pigments with the main structure of tripyrrole [12,13,14].

It has been verified that prodigiosins possess many healthcare functionalities, such as antibacterial, antimalarial, UV-protective and antineoplastic performances. Prodigiosins can endow fibers and fabric with added value when they are utilized as dyestuff [15,16,17]. However, some inherent shortages restrict their extensive application in the dyeing field. Firstly, microbial pigments can be classified into two generic groups: intracellular and extracellular pigments. As intracellular pigments, prodigiosins are almost insoluble in water. Before utilization, the pigments must be extracted from the interior of thalli by organic solvent. Besides, organic solvent is also indispensable for the preparation of dye liquid to dissolve the pigments. Furthermore, like most other microbial pigments, due to the low affinity between the pigment molecule and cellulose, dyeing cellulose fibers and fabric with prodigiosins faces huge challenges. Some papers report that cotton can merely be stained by prodigiosins without using chemical mordant, and the dyed cotton fabric does not have antibacterial activity against either *Escherichia coli* or *Staphylococcus aureus* due to the low dye-uptake [15,18,19].

In this study, a novel pH-mediated antimicrobial dyeing process of cotton fabric with microbial fermented prodigiosins nanomicelles was put forward. The preparation of dye liquor and the dyeing process entirely avoided the use of organic solvent and chemical mordant. In addition, dyed cotton fabric under the optimal process showed an outstanding antibacterial property. This research developed a widely-applicable and eco-friendly approach for antimicrobial intracellular pigments with the property of pH-sensitive solubility in water to endow cellulose fabric with color and antibacterial performance.

## 2. Materials and Methods

### 2.1. Materials

#### 2.1.1. Microorganism Material

*Serratia marcescens* ATCC 8100 was bought from American Type Culture Collection (Manassas, VA, USA).

#### 2.1.2. Textile Material

The scoured and bleached cotton fabric (weight 106.6 g/m^2^; warp 133 yarns per inch; weft 72 yarns per inch; thickness 0.21 mm) was bought from Tianyi printing and dyeing company in Tianjin, China.

#### 2.1.3. Chemical and Reagents

Standard prodigiosin was bought from Abcam company, Cambridge, UK. Peptone and yeast powder were of biological reagent. Tween 80, KCl, NaCl, MgSO_4_, glycerol and hydrochloric acid were of analytical reagent grade.

### 2.2. Preparation of Prodigiosins Nanomicelles Dye Liquor

The nanosuspension of prodigiosins micelles was prepared by the fermentation of *Serratia marcescens* through adding nonionic surfactant Tween 80 to the culture media in accordance with our previous paper [7]. The seed culture media was cultivated in a shaking incubator at 30 °C and 160 rpm for 24 h and then inoculated into the fermentation culture media, followed by cultivation at 28 °C and 200 rpm for 96 h. After fermentation, the cultivated bacteria solution was centrifuged at 10,000 rpm, 10 °C for 10 min to discard the thalli. The fermentation liquid obtained after centrifugation, namely the nanosuspension of prodigiosins micelles, was applied for the following dyeing experiment.

### 2.3. Dyeing Procedure

The prepared nanosuspension of prodigiosins micelles was used to dye cotton fabric. The pH of the dye bath was adjusted from 1 to 5 with 1 mol/L HCl solution, and the pH value spacing was 1. An Ahiba Duance Eco dyeing machine (Datacolor company, Lawrenceville, NJ, USA) was utilized for fabric dyeing, and each dyeing tank contained 5 g of cotton fabric and 100 mL of dye liquor. The dyeing process started from 30 °C with a rising velocity of 3 °C/min and a liquor ratio 1:20. After the heat preservation procedure, the dyeing machine cooled down with a descent velocity of 5 °C/min. The dye bath pH, dyeing temperature and dyeing time were investigated to determine the optimum dyeing condition. After the dyeing procedure, the fabrics were firstly washed with running water and then washed twice with boiling soap liquid for 10 min, followed by washing with running water and drying at 70 °C. The soap liquid contained 2 g/L soap flakes.

### 2.4. Measurements

#### 2.4.1. Particle Size Analysis

The DelsaNano C laser particle size analyzer (Beckman Coulter Inc., Brea, CA, USA) was utilized to measure the particle size distribution of the prodigiosin nanomicelles. The mean diameter of the particle size and the polydispersity index were obtained. The test was repeated three times.

#### 2.4.2. Scanning Electron Microscopy Observation

Water was removed from the nanosuspension of prodigiosins micelles by freeze-drying. The morphology and micro-structure of the prodigiosins nanoparticles were observed by a scanning electron microscopy (SEM, ZEISS Gemini 500, Carl Zeiss company, Oberkochen, Germany) at 10.0 kV. Prior to observation, the sample was coated with a thin layer of gold.

#### 2.4.3. Visible Light Scanning Analysis

The visible absorption spectrum (400–800 nm) was measured by a UV-3200 UV/Vis spectrophotometer (Mapada, Shanghai, China).

#### 2.4.4. Color Characteristics

The color strength (*K/S* value) and CIE L^*^, a^*^, b^*^, C^*^, h^*^ of dyed fabrics were measured by a Datacolor 600 spectrophotometer (Datacolor company, Lawrenceville, NJ, USA) under illuminant D65, by a 10° standard observer. The CIE system was a specialized color system designed by International Commission on illumination. The results were the average of eight consecutive tests at different positions. The *K/S* value was calculated by Equation (1):

*K/S* = (1 − *R*)^2^/2*R* (1)

where *R* is the observed reflectance of the dyed sample under the wavelength of 540 nm, *K* is the absorption coefficient and *S* is the scattering coefficient.

The colors are given in the CIELAB coordinate system: L^*^ presents luminosity; a^*^ presents redness–greenness (+value = red, −value = green); b^*^ presents yellowness–blueness (+value = yellow, −value = blue); C^*^ presents saturation; and h^*^ presents metric hue angle.

#### 2.4.5. Tensile Breaking Strength

The tensile breaking strength of all samples was tested by the YG065 electronic fabric strength tester (Laizhou Electron Instrument Co., Ltd, Laizhou, China) according to modified standard GB/T 3923.1-2013. The GB/T was recommended national standards of China. Sample width was 25 mm, and gauge length was 100 mm. The stretching speed was 100 mm/min. The result was the average of three specimens of each sample.

#### 2.4.6. XRD Analysis

XRD analysis of undyed and dyed cotton fabrics was conducted by a D/MAX-2500 X-ray diffractometer (Rigaku, Tokyo, Japan; Cu Kα, 40 kV, 100 mA) from 5° to 50° (2θ) at a scanning speed of 8°/min. Crystallinity of cotton was obtained by calculating the ratio of the crystalline phase area in the total area according to Equation (2), and this process was proceeded by MDI JADE software (6.5 version, Materials Data Inc., Livermore, CA, USA) through fitting the peaks of the XRD pattern.

Crystallinity = *A_cr_*/(*A_cr_* + *A_am_*) × 100% (2)

where *A_cr_* and *A_am_* are the diffraction peak area of the crystalline phase and the amorphous phase, respectively.

#### 2.4.7. Antibacterial Activity

The antibacterial experiment of dyed cotton was carried out according to the antibacterial standard for textiles (GB/T 20944.3-2008). Two selected test strains were *Escherichia coli* (*E. coli*, ATCC 8739, Gram-negative) and *Staphylococcus aureus* (*S. aureus*, ATCC 6538, Gram-positive). Small pieces of fabric sample (0.5 g) were introduced into the buffer solution (50 mL) which contained 2 × 10^4^–3.0 × 10^4^ colony forming units (CFU)/mL of bacteria, followed by shaking incubation at 24 °C for 18 h. Then, a 100-µL portion of each solution was seeded onto nutrient agar with a spread plate method after gradient dilution.

After the antibacterial test, a Scan 500 Colony Counter (Interscience, Saint Nom, France) was used to count bacteria colonies, and the reduction of bacteria was calculated according to Equation (3):

*R* (%) = (*A* − *B*)/*A* × 100% (3)

where *R* is the bacterial reduction rate (%) and *A* and *B* are the number of the visual bacterial colonies from undyed cotton and dyed cotton, respectively.

#### 2.4.8. Color Fastness

The rubbing, washing and perspiration colorfastness of dyed cotton were tested on the basis of ISO 105-X12, ISO 105-C10, ISO 105-E04, respectively.

## 3. Results and Discussion

**Figure 2:**
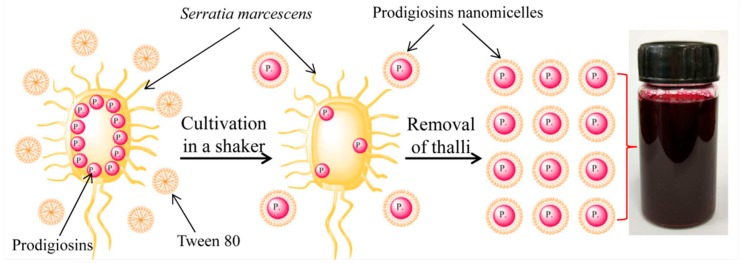
Preparation of prodigiosins nanosuspension.

As illustrated in Figure 2, the prodigiosins nanomicelles were prepared by the fermentation of *Serratia marcescens* through adding nonionic surfactant Tween 80 to the culture media. As a kind of intracellular pigment, most of the prodigiosins are located inside the *Serratia marcescens* cells in common nutrient solution. Nonionic surfactant Tween 80 could increase the permeability of membranes and cytoderm. As a consequence, the water-insoluble prodigiosins migrated away from the bacteria and wrapped into the micelles of surfactant Tween 80 under continuous oscillation [7,20,21]. After removal of thalli by high speed centrifugation, the nanosuspension of prodigiosins micelles was obtained.

### 3.1. Size Distribution of the Prodigiosins Nanomicelles

**Figure 3:**
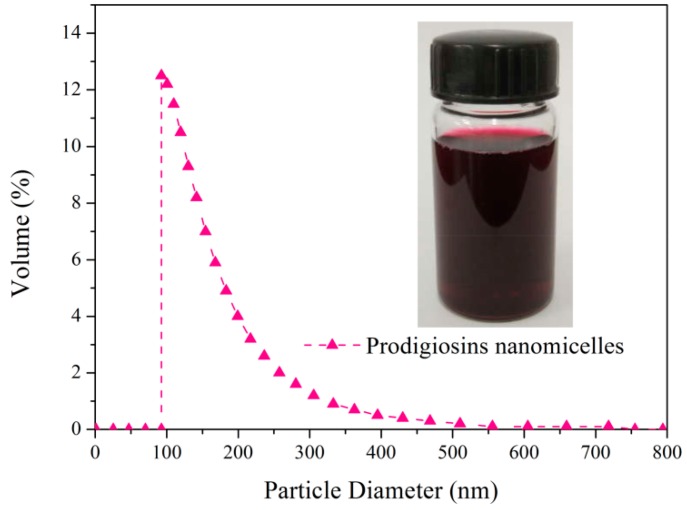
Particle size distribution of prodigiosins nanomicelles.

The particle size distribution of prodigiosins nanomicelles was tested to describe the dispersion system. As exhibited in Figure 3, the range of particle size was from 92.4 to 510.2 nm, and the average diameter of prodigiosins micelles was 222.3 nm. In addition, the polydispersity index was 0.343, which indicated that the size distribution of prodigiosins nanomicelles produced by microbial fermentation was relatively centralized. The nanoscale pigments ensured the uniformity and stability of the dispersion system, which could bring good levelness when dyeing fabric.

### 3.2. Surface Morphology of Prodigiosins Nanoparticles

**Figure 4:**
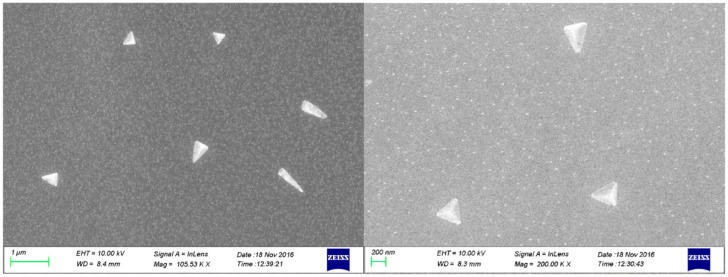
SEM images of prodigiosins nanoparticles produced by microbial fermentation.

Figure 4 shows SEM images of the prodigiosins nanoparticles with different magnifications. It was obvious that the nano-prodigiosins presented a tetrahedron shape, and the micromorphology of prodigiosins nanoparticles produced by microbial fermentation was first observed.

### 3.3. Visible Spectrum of Prodigiosins

**Figure 5:**
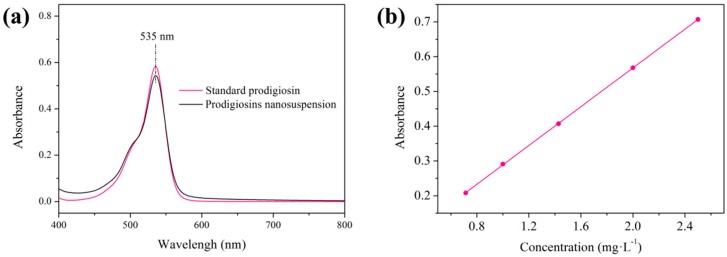
(**a**) Visible spectrum of standard prodigiosin and prodigiosins nanosuspension. (**b**) The fitting straight line of concentration/absorbance of standard prodigiosin solution.

It is known that the maximum absorption wavelength of prodigiosins appears at around 535 nm with a purplish red color under acidic and neutral condition [22,23]. The prodigiosins nanosuspension was diluted by 90% acid ethanol-water solution (pH 3), and the visible spectrum of the pigment solution was measured after removing insolubles by centrifugation. As shown in Figure 5a, the maximum absorption wavelength was observed at 535 nm, which was consistent with the standard prodigiosin.

The standard prodigiosin solutions (90% ethanol-water solution, pH 3) of different concentrations were prepared. Then, the absorbance/concentration relationship at the characteristic peak (535 nm) was established as illustrated in Figure 5b. The concentration of prodigiosins in the nanosuspension can be calculated according to the fitting straight line equation.

*Y* = 0.27884*X* + 0.00997, *R*^2^ = 0.99994 (4)

where *X* and *Y* are the values of concentration and absorbance, respectively.

By calculation, the concentration of prodigiosins in the nanosuspension was 76.46 mg/L.

### 3.4. Influence of Dye Bath pH on Dyeing Effect

Due to the presence of –NH– in the molecule, the solubility of prodigiosins in water is sensitive to pH value, which provides the basis for performing the pH-mediated dyeing process. The isoelectric point of prodigiosins is around pH 9.7. Although prodigiosins are almost insoluble in water under neutral condition, their solubility increases under acidic condition, especially in hot and acidic conditions [7,12,15]. During the dyeing process, the prodigiosins nanomicelles were broken down gradually, and the pigment inside was released followed by partial dissolution in the dye liquor under the acidic condition with high temperature. As a consequence, the prodigiosins with a high concentration on the fiber surface penetrated into cotton because of the concentration gradient.

Table 1 shows the apparent color and color characteristics of cotton fabrics dyed with the nanosuspension of prodiginines micelles under different pH values of 1–5. The initial dyeing temperature and dyeing time were set at 90 °C and for 60 min. It was evident that the color strength (*K/S* value) of dyed cotton increased gradually with the reduction of dye bath pH, which was because the solubility of prodigiosins increased in the more acidic condition. The dyed cotton fabric exhibited the maximum color strength when dye bath pH was 3. With further reduction of pH, the color strength decreased. Especially when the pH value was 1, the color became dim and shallow, which might be due to the damage of prodigiosins under the highly acidic and heating condition for a long duration. Therefore, the optimum dye bath pH was obtained at 3.

### 3.5. Effect of Dye Bath pH on Breaking Strength of Dyed Cotton Fabric

**Figure 6:**
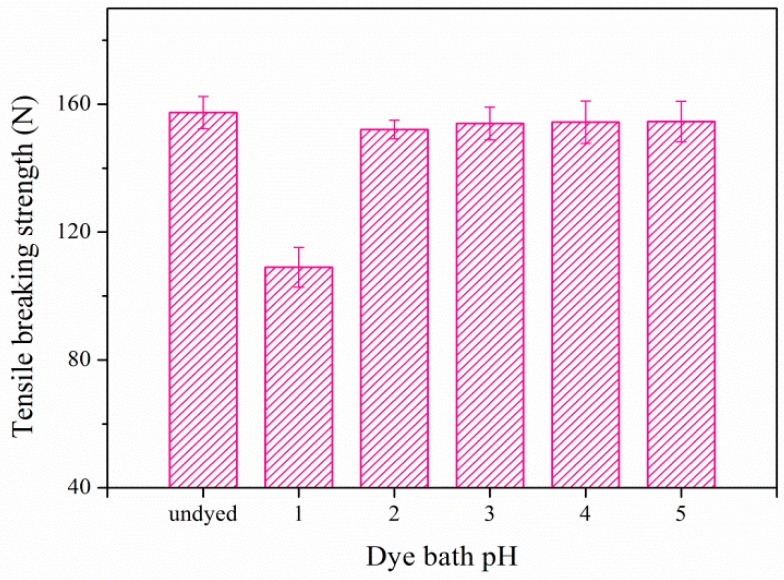
Effect of dye bath pH on tensile breaking strength of dyed cotton fabric.

It is well known that cotton is vulnerable in a highly acidic and heating condition, because of the hydrolysis of cellulose. As a result, the fabric strength may decrease in acidic dye liquor. The effect of dye bath pH on tensile breaking strength of dyed cotton fabrics is illustrated in Figure 6, which clearly showed that the tensile strength of dyed cotton under the optimal pH of 3 was almost the same as the undyed one. The error bars are standard deviations from three independent measurements. When the dye bath pH was 1, the tensile breaking strength decreased obviously. The result indicated that cotton fabric was not damaged during the dyeing process under the optimum pH of 3.

### 3.6. XRD Analysis of Cotton Fabric

**Figure 7:**
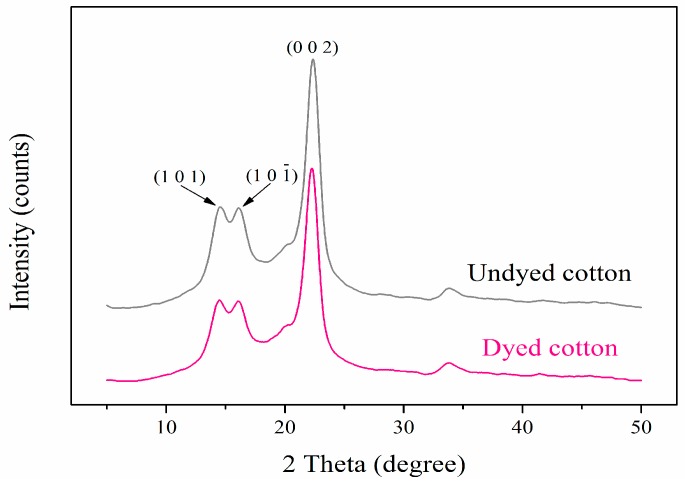
XRD patterns of undyed and dyed (pH 3.3) cotton fabrics.

The XRD patterns of undyed and dyed (pH 3) cotton fabrics are shown in Figure 7. Three obvious crystalline peaks could be observed in the pattern of undyed cotton and were labeled by the (1 0 1), (1 0 $\bar{1}$) and (0 0 2) planes at 2θ of 14.6°, 16.2° and 22.3°, respectively, which were characteristics of the cellulose I crystal structure [24,25]. It can be seen that the pattern of dyed cotton was almost identical to the undyed one, and the crystallinity of dyed (59.8%) cotton fabric was also quite close to the undyed (60.1%) cotton. Along with the breaking strength test, these results demonstrated that cotton almost suffered no damage through the dyeing process with the optimal pH of 3.

### 3.7. Effect of Dyeing Temperature and Dyeing Time on Color Strength

**Figure 8:**
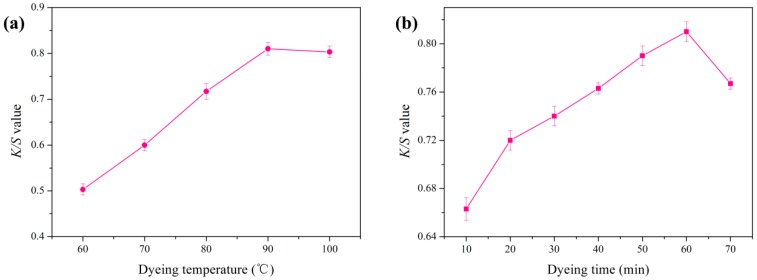
Effect of dyeing temperature (**a**) and dyeing time (**b**) on the *K/S* value of dyed cotton.

Under the optimum dye bath pH of 3, the most suitable dyeing temperature and dyeing time was explored. Figure 8a shows the effect of dyeing temperature on the *K/S* value of dyed cotton. The dyeing process was uniformly maintained for 60 min. It was clear that the color strength of dyed fabric increased gradually with the rise of dyeing temperature and reached the maximum at 90 °C. High temperature could enhance the dissolution of prodigiosins, which led to higher concentration of pigments on the fiber surface. As a result, it gained higher dye uptake and color strength.

The effect of dyeing time on the *K/S* value of dyed cotton under the condition of optimum pH 3 and dyeing temperature 90 °C is displayed in Figure 8b. A longer dyeing time resulted in a larger *K/S* value until the pigments on the surface and inside of the fibers reached equilibrium. When the dyeing process lasted for 60 min, the dyed cotton reached the maximum color strength. However, the color strength decreased with the further increase of dyeing time. The above phenomena might due to a part of the prodigiosins suffering damage under the long heating time, since microbial pigments possessed inferior thermostability compared to commercial synthetic dyestuff [26,27,28].

In conclusion, cotton fabric obtained the maximum *K/S* value when it was dyed with the nanosuspension of prodigiosins micelles under pH of 3 at 90 °C for 60 min. The optimum dyeing temperature and time corresponding to the parameters of the initial dyeing process.

### 3.8. Effect of Dye Bath pH on the Antibacterial Property of Dyed Cotton

**Figure 9:**
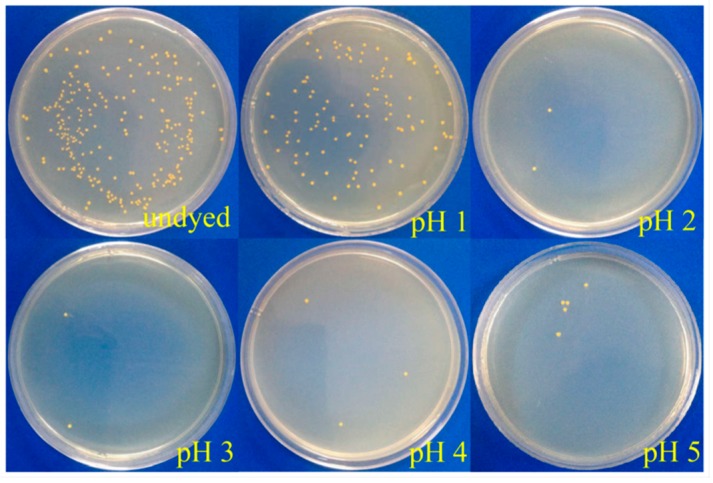
Antibacterial activity of cotton fabrics dyed under different pH values against *S. aureus*.

**Figure 10:**
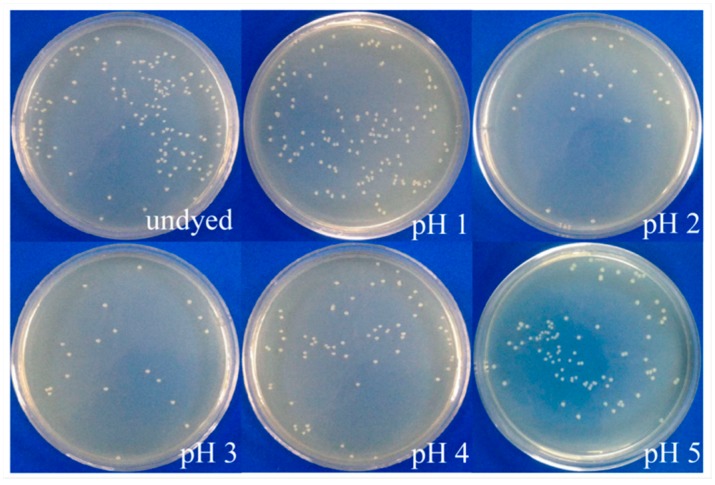
Antibacterial activity of cotton fabrics dyed under different pH values against *E. coli*.

It is acknowledged that prodigiosins possess excellent antibacterial activity toward many kinds of bacteria [29,30,31]. Figure 9 and Figure 10 show the influence of dye bath pH (1–5) on the antibacterial activities of dyed cotton fabrics against *S. aureus* and *E. coli*. It was found that the numbers of bacteria colonies from dyed cotton fabrics were distinctly less than those of the undyed one, and the reduction in the number of *S. aureus* colonies was more obvious than that of *E. coli* colonies.

**Figure 11:**
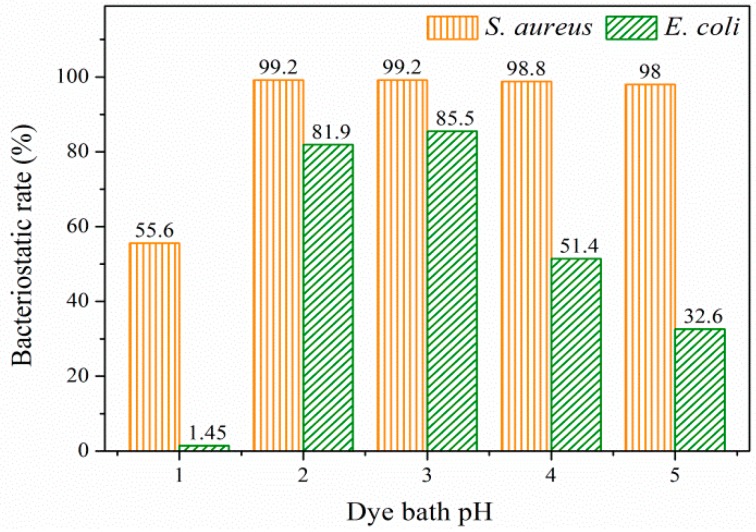
Bacteriostatic rates of cotton fabrics dyed under different pH values.

Figure 11 displays the bacteriostatic rates of cotton fabrics dyed under different pH values. It was obvious that the bacteriostatic rate echoed the color strength of dyed cotton, since the antibacterial activity was derived from prodigiosins. The maximum bacteriostatic rates of 99.2% against *S. aureus* and 85.5% against *E. coli* were observed when the dye bath pH was 3. When the dye bath pH was 1, dyed cotton exhibited almost no inhibition against *E. coli*, and its bacteriostatic rate against *S. aureus* was obviously decreased compared to other samples, which further demonstrated that the pigment was destroyed under pH of 1.

### 3.9. Color Fastness

The rubbing, washing and perspiration color fastness of dyed cotton under the optimal dyeing condition are displayed in Table 2. All the tested fastness ratings were above four, which indicated that the pH-mediated nanomicelles dyeing method endowed cotton fabric with good color fastness, so that dyed cotton could meet the wearing requirements very well, and its antimicrobial activity could be sustained during long time use. The antibacterial dyeing method in this paper could be generalized to other kinds of fabrics, such as wool, polyester and acrylic fabrics.

## 4. Conclusions

In this study, the novel pH-mediated process of dyeing cotton with prodigiosins nanomicelles realized dyeing and antimicrobial finishing simultaneously without organic solvent, chemical mordant and finishing agent. The results showed that when dye bath pH was 3, cotton fabric dyed under 90 °C for 60 min possessed the best dyeing effects with good rubbing, washing and perspiration fastness. In addition, the optimal antibacterial property of dyed cotton against *S. aureus* and *E. coli* was also obtained under pH 3.

The preparation of dye liquor and dyeing process in this paper provide a feasible and widely-applicable approach for antimicrobial intracellular pigments with the property of pH-sensitive solubility in water to endow cellulose fabric with color and antibacterial performance. It is worth noting that this antimicrobial dyeing process could be generalized to other cellulose materials applied in various fields, which call for antimicrobial function.

## Figures and Tables

**Table 1:** The effect of dye bath pH on apparent color, color strength and colorimetric parameters of dyed cotton fabrics.

Dye Bath pH	Apparent Color	*K/S* Value	L^*^	a^*^	b^*^	C^*^	h^*^
Undyed fabric	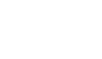	0.01	94.58	−0.28	2.36	2.38	96.78
1	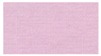	0.46	72.77	9.64	−4.28	10.55	336.08
2	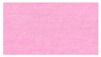	0.77	71.26	21.83	−9.08	23.64	337.42
3	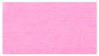	0.81	72.27	26.18	−10.10	28.06	338.90
4	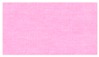	0.56	75.47	20.75	−8.02	22.25	338.87
5	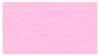	0.48	76.83	19.17	−6.26	20.17	341.92

**Table 2:** Color fastness of dyed cotton fabric.

Rubbing Fastness		Washing Fastness			Perspiration Fastness					
					Acid			Alkali		
Dry	Wet	CC	SC	SW	CC	SC	SW	CC	SC	SW
5	5	4–5	5	5	4–5	5	5	4	5	5

Color change (CC), staining on cotton fabric (SC) and staining on wool fabric (SW).
